# Hospital Resource Utilization and Patient Outcomes Associated with Respiratory Viral Testing in Hospitalized Patients 

**DOI:** 10.3201/eid2108.140978

**Published:** 2015-08

**Authors:** Sunita Mulpuru, Shawn D. Aaron, Paul E. Ronksley, Nadine Lawrence, Alan J. Forster

**Affiliations:** Ottawa Hospital Research Institute, Ottawa (S. Mulpuru, S.D. Aaron, N. Lawrence, A.J. Forster);; University of Ottawa, Ottawa, Ontario, Canada (S. Mulpuru, S.D. Aaron, A.J. Forster);; The Ottawa Hospital, Ottawa (S. Mulpuru, S.D. Aaron, A.J. Forster);; University of Calgary, Calgary, Alberta, Canada (P.E. Ronksley)

**Keywords:** nasopharyngeal swabs, respiratory viruses, infection control, isolation precautions, administrative data, viruses

## Abstract

Testing patients for respiratory viruses should guide isolation precautions and provide a rationale for antimicrobial drug therapies, but few studies have evaluated these assumptions. To determine the association between viral testing, patient outcomes, and care processes, we identified adults hospitalized with respiratory symptoms from 2004 through 2012 at a large, academic, tertiary hospital in Canada. Viral testing was performed in 11% (2,722/24,567) of hospital admissions and was not associated with reduced odds for death (odds ratio 0.90, 95% CI 0.76–1.10) or longer length of stay (+1 day for those tested). Viral testing resulted in more resource utilization, including intensive care unit admission, but positive test results were not associated with less antibiotic use or shorter duration of isolation. Results suggest that health care providers do not use viral test results in making management decisions at this hospital. Further research is needed to evaluate the effectiveness of respiratory infection control policies.

## Abstract 2

Results suggest that health care providers do not use viral test results in making management decisions.

In 2003, the coronavirus responsible for the severe acute respiratory syndrome (SARS) outbreak infected 774 and killed 8,096 persons worldwide (*1*). It was quickly recognized that this virus spread between close contacts, because 21% of infected case-patients were health care workers caring for patients infected with the SARS coronavirus (*1*,*2*). During the outbreak, respiratory infection control policies were developed by clinical infectious disease and public health experts, and their use was mandated in all Canadian hospitals. These measures were attributed to the eventual control of the outbreak (*3*–*6*). As a result, infection control practices, including strict hand hygiene, viral testing of patient samples, and use of isolation precautions, quarantine rooms, and personal protective equipment, were mandated for routine use with all patients who sought treatment at emergency departments (EDs) with respiratory symptoms and fever (*7*,*8*).

National guidelines suggest that patients admitted to acute care hospitals with infectious respiratory symptoms should receive screening for viral infections by answering symptom-based questionnaires, and they should be placed under droplet isolation precautions until definitive evidence rules out a transmissible respiratory illness (*7*,*9*). Viral testing in this setting is carried out with a nasopharyngeal (NP) swab sample, which is processed by direct fluorescent antibody (DFA), PCR, or both to identify a viral pathogen. Viral testing in these patients should improve diagnostic clarity, reduce the number of subsequent diagnostic tests and procedures required, and prevent infection transmission to other patients and health care workers by guiding the use of isolation precautions. However, these outcomes can only occur if physicians and infection control practitioners assess the results of the viral test and feel confident ruling out viral disease on the basis of the results.

To date, whether respiratory viral testing in patients improves outcomes or care processes has not been proven in large studies. Two small studies demonstrated that knowledge of the viral test results did not affect length of stay and subsequent antibiotic use (*10**,**11*). However, 1 previous study demonstrated reduced length of stay, mortality, and cost when using viral testing (*12*). These studies were limited by the following: relatively small sample sizes; only single winter seasons being evaluated; and utilization of hospital resources, including isolation precautions, not being assessed (*10*–*12*).

To address this gap in evidence, we set 2 main objectives for this study. First, we aimed to determine the association between the use of viral testing and subsequent hospital resource utilization (antibiotic/antiviral drugs prescribed; radiology studies conducted; cultures and bronchoscopies performed), including the duration of isolation precautions. Second, we aimed to determine whether viral testing was associated with in-hospital deaths, admission to intensive care, and length of stay in the hospital.

## Methods

### Data Sources

We conducted a large retrospective observational cohort analysis based at The Ottawa Hospital (TOH), an adult academic hospital located in Ottawa, Ontario, Canada, with ≈1,100 inpatient beds. TOH is a tertiary care referral center that provides care for 1.2 million patients in the Eastern Ontario region. We created the study cohort using hospital administrative and clinical data from The Ottawa Hospital Data Warehouse, a relational database containing information from TOH’s patient registration system, the clinical data repository (containing laboratory, pharmacy, radiology, and clinical notes), and the discharge abstract database. Data from these operational systems are loaded into the database on a daily basis and linked by patient unique identifiers. Extensive assessments of data quality were performed during the development of the database.

### Study Population: Inclusion and Exclusion Criteria

We identified hospitalizations of adult patients from January 1, 2004, through December 31, 2012. Hospitalizations were included if the patient was admitted through the ED with any combination of cough, fever, or shortness of breath.

We excluded hospitalizations resulting from a transfer from another health institution (such as a long-term care facility or another regional hospital). If a patient had been seen in the ED with respiratory symptoms but was not subsequently admitted, the patient was also excluded from the study.

### Exposure

The NP swab sample, processed by DFA or PCR, was the exposure of interest for each hospitalization. The standard of practice at our center during the study period was to process NP swab samples with DFA. However, during the 2009 influenza A(H1N1) pandemic season, multiplex PCR was used to detect viruses. The multiplex PCR can detect influenza A or B, respiratory syncytial virus, parainfluenza virus, enterovirus, adenovirus, human metapneumovirus, rhinovius, and coronavirus. We developed and validated an algorithm within our dataset to determine whether NP swab samples were analyzed, and categorized them as positive or negative on the basis of the DFA or PCR result (positive test refers to identification of a respiratory virus).

### Patient Outcomes and Measures of Health Resource Utilization

The primary outcomes considered in this study were number of inpatient deaths, and length of hospital stay. Secondary outcomes included admission to the intensive care unit (ICU) and measures of resource utilization, including antibiotic and antiviral prescriptions, chest radiograph and computed tomography imaging, blood and sputum cultures, bronchoscopy, and use and duration of isolation precautions in the hospital.

### Analysis and Adjustment for Confounding

The unit of analysis in this study was the patient’s hospitalization. Patient characteristics were compared across groups (with and without viral testing performed) and were described by using proportions, means with SDs, and medians with interquartile range when appropriate. Using similar methods, we then compared groups with positive and negative NP swab sample results among the hospitalizations in which an NP swab sample was analyzed. We assessed the difference of means and SD for continuous variables using a 1-way analysis of variance test (ANOVA) and for differences between proportions using a χ^2 ^test. For all statistical tests, p<0.05 was considered statistically significant.

We measured patient coexisting conditions using the Elixhauser score (*13*,*14*), a validated scoring system which summarizes comorbid illness and can predict the patient’s risk of death in the hospital (*14*). It was derived and validated by using hospitalization data from TOH, and the score was based on the original 30 comorbidity diagnosis groups in the Elixhauser comorbidity classification system (*13*,*14*). The Elixhauser summary score ranges from a minimum of −19 to +89, which are associated, respectively, with a 0.37% and 99.41% risk of in-hospital death (*14*). Baseline risk of death at the time of hospitalization was calculated for each hospitalization by using a regression model previously validated by data from TOH’s patient population (*15*,*16*). We defined influenza seasons on the basis of dates recorded in the Public Health Agency of Canada’s national surveillance system for influenza, FluWatch (*17*). We used this information to categorize hospital admissions according to whether or not they occurred during an influenza season.

We created multivariate logistic regression models to investigate whether having an NP swab sample obtained and tested was associated with probability of death and ICU admission. For each outcome in which the patient died or was admitted to the ICU, univariate odds ratios (ORs) were calculated for patient sociodemographic factors, clinical factors related to the hospitalization, patient comorbidity, and whether the patient was admitted to the hospital during influenza season. A multivariate model was created on the basis of significant predictors of death and ICU admission and was reduced by using stepwise variable selection. We used unadjusted and adjusted linear regression models to determine the change in length of stay in hospital when an NP swab sample was tested during the admission process. We used a natural logarithm transformation of the length of stay variable to adjust for the left skewed distribution of this variable.

In a secondary analysis, the same methods were used to develop multivariate logistic regression models to investigate the association between the NP swab sample testing (positive or negative test result) and odds of death and ICU admission. Multivariate linear regression was used to evaluate length of stay.

We conducted a sensitivity analysis of a subgroup of hospitalizations in which the most responsible discharge diagnosis was a pulmonary infection or exacerbation. This was done to account for the fact that patients seeking treatment for respiratory symptoms could have received a diagnosis of a noninfectious condition (such as heart failure or pulmonary embolism). We limited the discharge diagnosis to diagnoses of respiratory infections or exacerbations to determine whether there was any effect on the study outcomes. In this group, we assessed the potential association between having an NP swab sample tested and clinical outcomes (Technical Appendix Table 1). All analyses were conducted by using SAS 9.2 statistical software (SAS Institute Inc., Cary, NC, USA). This study was approved by the Ottawa Health Sciences Network Research Ethics Board, and a waiver of patient consent was granted.

## Results

### Study Cohort Demographics

During the 8-year study period, we identified 24,567 hospital admissions in which the patient sought treatment at the ED reporting chief symptoms of fever and/or cough and/or shortness of breath. These admissions represented 17,327 unique patients. Baseline characteristics of the study cohort are described in Table 1. An NP swab sample was tested in 11% (2,722/24,567) of admissions. Overall, patients who had an NP swab sample tested were younger, more likely to be admitted during influenza season, and more likely to be female.

**Table 1:** Baseline characteristics of hospitalized adults admitted with respiratory symptoms, Ottawa, Ontario, Canada, 2004–2012*

Variable	Without NP swab samples, n = 21,845	With NP swab samples, n = 2,722	Total, n = 24,567	p value
Age at admission, mean ± SD	67.7 ± 17.14	65.99 ± 18.31	67.48 ± 17.28	<0.001
Female sex, no. (%)	10,891 (49.9)	1,420 (52.2%)	12,311 (50.15)	0.023
Admitted during influenza season, no. (%)	12,958 (59.3)	2,221 (81.6%)	15,179 (61.8)	<0.001
Baseline risk of death, mean ± SD	0.14 ± 0.15	0.14 ± 0.14	0.14 ± 0.15	0.65
Elixhauser score quartiles				<0.001
0	5,636 (25.8)	981 (36.0)	6.617 (26.9)	
1	5,877 (26.95)	762 (28.0)	6,639 (27.0)	
2	5,200 (23.8)	558 (20.55)	5,758 (23.4)	
3	5,132 (23.5)	421 (15.5)	5,553 (22.6)	

*NP, nasopharyngeal.

### Description of Outcomes

Table 2 describes likelihood of deaths, ICU admission, length of stay, and use of isolation precautions in the study cohort and among hospitalizations in which the patient had a positive or negative NP swab sample. During hospitalizations in which an NP swab sample was tested, length of stay in hospital was longer (11.7 days vs. 9.5 days, p<0.001) and mean duration of isolation precautions was longer (4.8 days vs. 1.4 days, p<0.001) than in hospitalizations in which an NP swab sample was not tested. There was no significant difference in the mean number of days spent in isolation between hospitalizations in which the patient had positive or negative NP swab samples (5.16 ± 5.39 vs. 4.73 ± 7.65 days, p = 0.27).

**Table 2:** Outcomes for hospitalized adults seeking treatment with respiratory symptoms, Ottawa, Ontario, Canada, 2004–2012*

Outcome variable	Study cohort, n = 24,567	With negative swab sample, n = 2,302	With positive swab sample, n = 420	p value†
Death, no. (%)	2,550 (10.4)	239 (10.4)	40 (9.5)	0.594
ICU admission, no. (%)	2,007 (8.2)	341 (14.8)	76 (18.1)	0.086
Days in ICU, mean ± SD	8.37 ± 10.64	11.22 ± 12.77	11.70 ± 14.03	0.771
Hospital isolation used, no. (%)	7,487 (30.5)	1,993 (86.6)	396 (94.3)	<0.001
No. days in isolation, mean ± SD	1.79 ± 6.79	4.73 ± 7.65	5.16 ± 5.39	0.27

*ICU, intensive care unit. †For negative and positive swab samples.

### NP Swab Samples and Resource Utilization

Table 3 describes the use of hospital resources (antibiotic drugs, antiviral drugs, chest imaging studies, cultures, and bronchoscopy) among hospitalized patients with positive and negative NP swab samples. Among hospitalizations in which the sample was positive (420/2,722) and hospitalizations in which it was negative (2,302/2,722), no significant differences were found in process of care variables, with exception of more antiviral drug use and less use of computed tomography chest scans in the group with positive swab samples. Hospitalizations in which an NP swab sample was analyzed used statistically more resources than those in which no swab sample was tested (p<0.001, for all hospital resources measured).

**Table 3:** Laboratory, prescription, radiology, and procedure use among hospitalized patients with positive and negative NP swab samples, Ottawa, Ontario, Canada, 2004–2012*

Variable	No. (%) negative NP swab samples, n = 2,302	No. (%) positive NP swab samples, n = 420	No. (%) total swab samples, N = 2,722	p value
Antibiotic use	2,204 (95.7)	397 (94.5)	2,601 (95.6)	0.265
Antiviral use	305 (13.2)	166 (39.5)	471 (17.3)	<0.001
Blood cultures	1,813 (78.8)	340 (81.0)	2153 (79.1)	0.309
Sputum cultures	979 (42.5)	167 (39.8)	1146 (42.1)	0.291
Bronchoscopy	147 (6.4)	20 (4.8)	167 (6.1)	0.202
CT scan of thorax	599 (26.0)	83 (19.8)	682 (25.1)	0.006
Chest radiograph	1,293 (56.2)	229 (54.5)	1,522 (55.9)	0.532

*NP, nasopharyngeal; CT, computed tomography.

### NP Swab Samples and Association with Odds of Death, ICU Admission, and Length of Stay

After adjustment for confounding variables, there was no association between having an NP swab sample tested in the hospital and odds of death (OR 0.90, 95% CI 0.76–1.10). We identified a significant increase in ICU admission when a patient’s NP swab sample had been tested (OR 2.23, 95% CI 1.61–3.10). Finally, linear regression analysis demonstrated a nonsignificant 1-day increase in length of stay among hospitalized patients for whom a sample was tested (p = 0.55; ORs with 95% CIs are shown in Technical Appendix Table 2). Among the hospitalizations in which an NP swab sample was tested (n = 2,722), no significant associations were found between a positive swab sample and odds of death, ICU admission, or length of stay (Technical Appendix Table 3).

### Results of Sensitivity Analysis

In a restricted cohort of hospitalized patients in which an infectious respiratory diagnosis was made (n = 7,459), the fact that an NP swab specimen was tested was not associated with reduction in chance of death but was significantly associated with increased ICU admission. Length of stay was also significantly increased by 1 day (95% CI 0.9–1.1 days, p = 0.04), which was not the case in the primary analysis (Technical Appendix Table 1).

## Discussion

In this study, viral testing of respiratory samples during hospitalization was not associated with a significant reduction in odds of patient deaths or length of hospital stay after adjustment for critical clinical confounding factors. Viral testing, however, was associated with increased likelihood of admission to the ICU. Our study also did not find that respiratory viral testing was associated with significant reductions in antibiotic use, chest imaging studies, bronchoscopy, or microbiologic cultures among patients with infectious respiratory symptoms. Most notably, a positive viral test result did not lead to significant reductions in antibiotic use, number of chest radiographs obtained, and number of blood cultures requested. It is plausible that lack of any observable beneficial effect on these outcomes is a result of health care providers neglecting to adjust care processes on the basis of the testing results.

Although more isolation precautions were used with patients with positive viral test results than with those with negative test results (94% vs. 87%, p<0.001), the test result did not influence the duration of isolation precautions. No statistical difference was found in the mean number of isolation-days between hospitalizations in which positive and negative viral test results were obtained (5.2 days vs. 4.7 days, p = 0.27). 

This finding could have several potential causes, however. First, health care providers may not be translating negative test results into the action of removing isolation precautions because of lack of infection control directives for front-line staff (nurses and physicians) to guide the safe removal of isolation precautions. As a result, patients may remain under isolation precautions for a standard fixed duration, regardless of the viral test result. Second, perhaps front-line staff fear the possibility of infection transmission (even when the NP swab sample is negative) and continue the precautions as a conservative measure.

We found that hospitalized patients for whom NP swab samples were tested had a greater chance of ICU admission, after adjustment for confounders, including admission during influenza season, isolation status, and baseline risk for death. This observation remains unexplained. It may be due to residual confounding, but it is also conceivable that obtaining the NP swab sample and subsequent isolation precautions may put some patients at risk for adverse events that require ICU admission. Abad et al. conducted a systematic review and found that isolation precautions are associated with greater adverse drug events, less physician and nurse care, and increased patient scores for anxiety and depression (*18*). In a prospective study, Stelfox et al. found that patients in isolation experienced more preventable adverse events in the hospital, made more formal complaints to the hospital about their care, were more likely to have had no vital signs done when ordered, and had more days with no physician progress notes, when compared with nonisolated controls (*19*).

Relatively few studies have evaluated the effects of respiratory viral testing on processes of care and clinical outcomes in hospitalized adults. Most of these studies were performed on children in the ED (*20*–*23*). However, 2 small prospective studies in adult patients have examined the effects of respiratory viral testing results on antibiotic use, whereas 1 other study also examined the effect on length of hospital stay (*10*,*11*). Hernes et al. prospectively studied the effect of respiratory viral PCR testing on antibiotic treatment and length of stay among 147 hospitalized patients >65 years of age with respiratory infections and found no difference in antibiotic use or length of stay between patients with a positive and negative viral test result (*10*). An earlier study in 2005 by Oosterheert et al. found similar results among 107 adult patients admitted for antibiotic treatment of lower respiratory tract infection (*11*). Their overall conclusion was that early knowledge of the viral test result did not significantly reduce the duration of antibiotics, or costs, when compared with those of a group in which the viral test results were not made available (*11*).

The results of these 2 small prospective studies are generally congruent with our results. However, we also found that a positive viral test result did not affect other processes of care, including whether blood and sputum cultures, bronchoscopy, and chest radiographs were obtained and, most notably, duration of isolation precautions. Our study also examined the outcomes of patient death and ICU admission, which were not addressed in the previous studies.

Our study has several strengths. It is the largest study conducted to evaluate the effects of respiratory viral testing on clinical outcomes in adult hospitalized patients. Also, our data spanned 8 years, including 8 seasons of viral infections. Given our sample size, all adjusted regression models had adequate power to evaluate the chance of death and ICU admission outcomes (*24*).

Our study also has several limitations. The retrospective nature of this study makes the results vulnerable to unmeasured confounding. We accounted for temporal confounding due to influenza seasonality and for confounding by indication using validated measures of baseline mortality risk and comorbidity in the adjusted regression models. However, we did not capture acute vital signs and other nonlaboratory clinical data at the time patients sought treatment, which may have influenced the outcomes we studied. Finally, the study was conducted by using data from a single academic center, which has implications for the generalization of these results to other medical institutions. However, the tertiary care hospital in this study follows national and international recommendations for infection control practices, which reduces the likelihood that practices would be significantly different from other major medical centers.

Our results suggest that in this academic center during the study period, respiratory viral testing did not achieve the goals of reducing antibiotic prescriptions and other diagnostic tests, nor did it result in timely discontinuation of isolation precautions. Because the duration of isolation is not guided by the viral test result, one questions whether the process of viral testing is helping reduce viral infection transmission in the hospital. We could not assess infection transmission in this study, but future work is required in this area.

Our findings should encourage hospital administrators and infection control practitioners to reevaluate current practices, so that viral test results are used appropriately to modify subsequent treatments and guide provision of isolation precautions. This study sets the foundation for further research to ensure that current policies and practices result in efficient resource utilization and prevent infection transmission in hospitals.

**Technical Appendix.** 4 tables showing the following: odds of death and ICU admission among hospitalized adults for whom an NP swab sample was analyzed, in a subgroup of hospitalizations in which the most responsible discharge diagnosis was a respiratory infection or exacerbation; odds of death and ICU admission among hospitalized adults when an NP swab sample was analyzed; adjusted regression models evaluating the association between a positive NP swab result and death, ICU admission, and length of stay; and predictor variables used in the multivariate logistic and linear regression models.

## References

[1] World Health Organization Global Alert and Response. Summary of probable SARS cases with onset of illness from 1 November 2002 to 31 July 2003 [cited 2014 May 22]. http://www.who.int/csr/sars/country/table2004_04_21/en/

[2] Lapinsky SE. Epidemic viral pneumonia. Curr Opin Infect Dis. 2010;23:139–44. 10.1097/QCO.0b013e328336eaae 20087179

[3] Ontario Ministry of Health and Long Term Care. Preventing respiratory illnesses: protecting patients and staff. Recommended infection control and surveillance standards for febrile respiratory illness (FRI) in non-outbreak conditions [cited 2014 May 22]. http://www.health.gov.on.ca/fr/public/programs/emu/sars/reports/dir_122303_acute_care_nonoutbreak.pdf

[4] Dwosh HA, Hong HHL, Austgarden D, Herman S, Schabas R. Identification and containment of an outbreak of SARS in a community hospital. CMAJ. 2003;168:1415–20 . 12771070 PMC155957

[5] Shaw K. The 2003 SARS outbreak and its impact on infection control practices. Public Health. 2006;120:8–14. 10.1016/j.puhe.2005.10.002 16297415 PMC7118748

[6] Bridges CB, Kuehnert MJ, Hall CB. Transmission of influenza: implications for control in health care settings. Clin Infect Dis. 2003;37:1094–101. 10.1086/378292 14523774

[7] Public Health Agency of Canada. Routine practices and additional precautions for preventing the transmission of infection in healthcare settings, 2012 [2014 May 22]. http://www.ipac-canada.org/pdf/2013_PHAC_RPAP-EN.pdf

[8] Centers for Disease Control and Prevention. Seasonal influenza [cited 2014 May 22]. http://www.cdc.gov/flu/about/disease/us_flu-related_deaths.htm#how-many-die?

[9] Public Health Ontario. Best practices for infection prevention and control programs in Ontario in all health care settings. 3rd ed. 2012 [cited 2014 May 22]. http://www.publichealthontario.ca/en/eRepository/BP_IPAC_Ontario_HCSettings_2012.pdf

[10] Hernes SS, Hagen E, Quarsten H, Bjorvatn B, Bakke PS. No impact of early real-time PCR screening for respiratory viruses on length of stay and use of antibiotics in elderly patients hospitalized with symptoms of a respiratory tract infection in a single center in Norway. Eur J Clin Microbiol Infect Dis. 2014;33:359–64. 10.1007/s10096-013-1963-0 23999830 PMC7088319

[11] Oosterheert JJ, van Loon AM, Schuurman R, Hoepelman AI, Hak E, Thijsen S, Impact of rapid detection of viral and atypical bacterial pathogens by real-time polymerase chain reaction for patients with lower respiratory tract infection. Clin Infect Dis. 2005;41:1438–44. 10.1086/497134 16231254 PMC7107964

[12] Barenfanger J, Drake C, Leon N, Mueller T, Troutt T. Clinical and financial benefits of rapid detection of respiratory viruses: an outcomes study. J Clin Microbiol. 2000;38:2824–8 . 10921934 10.1128/jcm.38.8.2824-2828.2000 PMC87120

[13] Elixhauser A, Steiner C, Harris DR, Coffey RM. Comorbidity measures for use with administrative data. Med Care. 1998;36:8–27. 10.1097/00005650-199801000-00004 9431328

[14] van Walraven C, Austin PC, Jennings A, Quan H, Forster AJ. A modification of the Elixhauser comorbidity measures into a point system for hospital death using administrative data. Med Care. 2009;47:626–33. 10.1097/MLR.0b013e31819432e5 19433995

[15] van Walraven C, Escobar GJ, Greene JD, Forster AJ. The Kaiser Permanente inpatient risk adjustment methodology was valid in an external patient population. J Clin Epidemiol. 2010;63:798–803. 10.1016/j.jclinepi.2009.08.020 20004550

[16] Escobar GJ, Greene JD, Scheirer P, Gardner MN, Draper D, Kipnis P. Risk-adjusting hospital inpatient mortality using automated inpatient, outpatient, and laboratory databases. Med Care. 2008;46:232–9. 10.1097/MLR.0b013e3181589bb6 18388836

[17] Public Health Agency of Canada. FluWatch Canada, 2013 [2014 May 22]. http://www.phac-aspc.gc.ca/fluwatch/13-14/index-eng.php

[18] Abad C, Fearday A, Safdar N. Adverse effects of isolation in hospitalised patients: a systematic review. J Hosp Infect. 2010;76:97–102. 10.1016/j.jhin.2010.04.027 20619929 PMC7114657

[19] Stelfox HT, Bates DW, Redelmeier DA. Safety of patients isolated for infection control. JAMA. 2003;290:1899–905. 10.1001/jama.290.14.1899 14532319

[20] Bonner AB, Monroe KW, Talley LI, Klasner AE, Kimberlin DW. Impact of the rapid diagnosis of influenza on physician decision-making and patient management in the pediatric emergency department: results of a randomized, prospective, controlled trial. Pediatrics. 2003;112:363–7. 10.1542/peds.112.2.363 12897288

[21] Iyer SB, Gerber MA, Pomerantz WJ, Mortensen JE, Ruddy RM. Effect of point-of-care influenza testing on management of febrile children. Acad Emerg Med. 2006;13:1259–68. 10.1111/j.1553-2712.2006.tb00287.x 17079787

[22] Wishaupt JO, Russcher A, Smeets LC, Versteegh FGA, Hartwig NG. Clinical impact of RT-PCR for pediatric acute respiratory infections: a controlled clinical trial. Pediatrics. 2011;128:e1113–20. 10.1542/peds.2010-2779 21987698

[23] Özkaya E, Cambaz N, Coşkun Y, Mete F, Geyik M, Samanci N. The effect of rapid diagnostic testing for influenza on the reduction of antibiotic use in paediatric emergency department. Acta Paediatr. 2009;98:1589–92. 10.1111/j.1651-2227.2009.01384.x 19555447

[24] Peduzzi P, Concato J, Kemper E, Holford TR, Feinstein AR. A simulation study of the number of events per variable in logistic regression analysis. J Clin Epidemiol. 1996;49:1373–9 . 10.1016/S0895-4356(96)00236-3 8970487

