# Supplementary material for: Hospital Resource Utilization and Patient Outcomes Associated with Respiratory Viral Testing in Hospitalized Patients
Source: Emerg Infect Dis. 2015 Aug;21(8):1366–71. doi: 10.3201/eid2108.140978 (PMC4517710; doi:10.3201/eid2108.140978)
Supplement: Supplementary file 1 — Technical Appendix. 4 tables showing the following: odds of death and ICU admission among hospitalized adults for whom an NP swab sample was analyzed, in a subgroup of hospitalizations in which the most responsible discharge diagnosis was a respiratory infection or exacerbation; odds of death and ICU admission among hospitalized adults when an NP swab sample was analyzed; adjusted regression models evaluating the association between a positive NP swab result and death, ICU admission, and length of stay; and predictor variables used in the multivariate logistic and linear regression models. [file 14-0978-Techapp-s1.pdf]

Article DOI: http://dx.doi.org/10.3201/eid2108.140978

Hospital Resource Utilization and Patient Outcomes Associated with Respiratory Viral Testing in Hospitalized Patients

Technical Appendix

Technical Appendix Table 1. Odds of death and ICU admission among hospitalized adults for whom an NP swab sample was analyzed, in a subgroup of hospitalizations in which the most responsible discharge diagnosis was a respiratory infection or exacerbation (n = 7459)

Outcome	Adjusted odds ratio (95% CI)
Death	0.84 (0.66–1.08)
ICU admission	
Effect of viral test†	2.63 (1.72–4.03)
Effect of test and isolation†	1.10 (0.86–1.41)
Effect of isolation†	1.22 (1.00 – 1.50)
	Adjusted no. days (95% CI)
Length of stay†	1.05 (0.904–1.102), p = 0.04

*ICU, intensive care unit; NP, nasopharyngeal.
There was a significant interaction term between isolation status and whether an NP swab sample was tested in the regression model predicting ICU admission. Effects of the interaction term and individual components are denoted with a dagger.
‡Indicates linear regression model used to determine change in length of stay when an NP swab sample was tested.

Technical Appendix Table 2. Odds of death and ICU admission among hospitalized adults when an NP swab sample was analyzed. Variables included in the adjusted models are listed in Technical Appendix Table 4.

Outcome	Adjusted odds ratio (95% CI)
Death	0.90 (0.76–1.10)
ICU admission	2.23 (1.61–3.10)
Effect of viral test†	
Effect of test and isolation†	1.52 (1.30–1.79)
Effect of Isolation†	1.44 (1.28–1.62)
	Adjusted no. days (95% CI)
Length of stay‡	0.99 (0.95–1.03), p = 0.55

*NP, nasopharyngeal.
†There was a significant interaction term between isolation status and whether an NP swab sample was tested in the regression model predicting ICU admission. Effects of the interaction term and individual components are denoted with a dagger.
‡Indicates linear regression model used to determine change in length of stay when an NP swab sample was tested.

Technical Appendix Table 3. Adjusted regression models evaluating the association between a positive NP swab result and death, ICU admission, and length of stay (n = 2,722)

Outcome	Adjusted odds ratio (95% CI)
Death	0.89 (0.60–1.32)
ICU admission	0.97 (0.70–1.33)
	Adjusted no. days (95% CI)
Length of stay†	1.00 (0.90–1.10); p value = 0.98

*NP, nasopharyngeal; ICU, intensive care unit.
†Indicates linear regression model used to determine change in length of stay when an NP swab sample was positive.

Technical Appendix Table 4. Predictor variables used in the multivariate logistic and linear regression models*

Variable	Death outcome	ICU admission outcome	Length of stay outcome
Viral test status	√	√	√
Baseline risk for death (at admission)	√	√	√
Admission during influenza season	√	√	√
Isolation status	√	√	√
Age	√	√	√
Antibiotic use	√	√	√
Antiviral use	√	√	√
Chronic pulmonary disease	√	√	
Congestive heart failure			√
Metastatic cancer	√	√	√
Solid tumor without metastasis	√		
Complicated diabetes	√	√	√
Renal disease		√	
Viral test status*isolation status (interaction term)		√	
ICU admission			√

*ICU, intensive care unit.
